# Increased Variation in Body Weight and Food Intake Is Related to Increased Dietary Fat but Not Increased Carbohydrate or Protein in Mice

**DOI:** 10.3389/fnut.2022.835536

**Published:** 2022-03-08

**Authors:** Yingga Wu, Sumei Hu, Dengbao Yang, Li Li, Baoguo Li, Lu Wang, Min Li, Guanlin Wang, Jianbo Li, Yanchao Xu, Xueying Zhang, Chaoqun Niu, John R. Speakman

**Affiliations:** ^1^State Key Laboratory of Molecular Developmental Biology, Institute of Genetics and Developmental Biology, Chinese Academy of Sciences, Beijing, China; ^2^University of Chinese Academy of Sciences, Beijing, China; ^3^Institute of Biological and Environmental Sciences, University of Aberdeen, Aberdeen, United Kingdom; ^4^Beijing Advanced Innovation Center for Food Nutrition and Human Health, Beijing Engineering and Technology Research Center of Food Additives, National Soybean Processing Industry Technology Innovation Center, Beijing Technology and Business University, Beijing, China; ^5^Shenzhen Key Laboratory of Metabolic Health, Center for Energy Metabolism and Reproduction, Shenzhen Institutes of Advanced Technology, Chinese Academy of Sciences, Shenzhen, China; ^6^University of Dali, Dali, China; ^7^CAS Center of Excellence in Animal Evolution and Genetics, Kunming, China

**Keywords:** protein, fat, carbohydrate, mice, strain, variation

## Abstract

A variety of inbred mouse strains have been used for research in metabolic disorders. Despite being inbred, they display large inter-individual variability for many traits like food intake and body weight. However, the relationship between dietary macronutrients and inter-individual variation in body weight and food intake of different mouse strains is still unclear. We investigated the association between macronutrient content of the diet and variations in food intake, body composition, and glucose tolerance by exposing five different mouse strains (C57BL/6, BALB/c, C3H, DBA/2, and FVB) to 24 different diets with variable protein, fat, and carbohydrate contents. We found only increasing dietary fat, but not protein or carbohydrate had a significant association (positive) with variation in both food intake and body weight. The highest variation in both body weight and food intake occurred with 50% dietary fat. However, there were no significant relationships between the variation in fat and lean mass with dietary protein, fat, or carbohydrate levels. In addition, none of the dietary macronutrients had significant impacts on the variation in glucose tolerance ability in C57BL/6 mice. In conclusion, the variations in food intake and body weight changes increased with the elevation of dietary fat levels.

## Introduction

Obesity is a major worldwide health issue. Obesity increases the risk of many chronic diseases, including type 2 diabetes, cardiovascular diseases, hypertension, and cancer (1). There is a continuous debate on how food macronutrient composition relates to body weight control (2, 3). It is still uncertain whether high-fat, high-glycemic-index carbohydrates, including sugar, low protein, or all the three macronutrients, are the cause of the elevated energy intake and obesity in humans (4–6). However, in mice, we have established that only an increased dietary fat content was associated with an elevated energy intake and adiposity by exposing 5 different mouse strains (C57BL/6, BALB/c, C3H, DBA/2, and FVB) to 29 different diets with varying protein, fat, and carbohydrate contents (7).

Despite being inbred, mice fed with high-fat diets display large individual variations in weight gain (8–10). Several studies have indicated that different mouse strains also differ in their physiological phenotype when treated with a high-fat diet (11–14). There is debate, however, about whether specific mouse strains should be classed as obesity-prone or obesity-resistant. For example, FVB and DBA/2 mouse strains have been described as both obesity-prone and obesity-resistant by different laboratories (13, 15). The C57BL/6 mouse strain has been suggested to be the best strain for studying metabolic diseases, such as obesity and type 2 diabetes (16, 17). It is consistently described as “obesity prone”; however, it can be defined as either “diabetic prone” or “diabetic resistant” depending on which sub-strain was used (18). This strain also shows a considerable non-genetic-related variation in body weight gain when fed with a high-fat diet (19, 20). Several studies have investigated the potential mechanism related to variation in weight gain when fed with high-fat diets. Diet-induced obese mice had increased hypothalamic orexigenic and decreased anorexigenic neuropeptide gene expressions compared to diet-resistant mice when fed with a high-fat diet in the C57BL/6 mouse strain (21, 22). Furthermore, it has been recently shown that the inter-individual variability for high-fat intake in C57BL/6 mice was linked to dopamine neuron activity (23). These non-genetic variations in later-life responses to a high-fat diet seem to stem from the early-life environment of the individual mice, in particular the litter size they were raised in and hence their early-life nutritional status (15, 24).

Increased adiposity is linked to the higher risk of the development of type 2 diabetes (25). However, elevated adiposity is not inevitably linked to metabolic dysfunction (26, 27). For example, there is a population of people who have obesity but are metabolically healthy (27). In addition, it has been indicated in mice that 50% of mice became obese and diabetic, 10% lean and diabetic, 10% lean and non-diabetic, and 30% showed intermediate phenotype after being fed with a high-fat diet for 9 months (28). In our own studies, however, glucose tolerance was strongly linked to changes in body mass and fatness, but there was considerable residual variation at any given level of adiposity that was not related to the diet (29). Such variation in insulin resistance and glucose production in C57BL/6 and AKR mouse strains has been related to the differential expression of GLUT4 protein in adipose tissue (30).

All the work, thus far, on non-genetic variation in body mass and food intake has concerned the responses of mice to high-fat diets (29). It is interesting to know the extent to which the variation observed in response to high-fat diets is also observed in response to the intake of other macronutrients. Do mice, e.g., show an elevated variation in food intake and body weight when fed with low protein, or diets with high levels of high-glycemic-index carbohydrates. We have previously studied the responses of mice to a matrix of 24 different diets with varying protein, fat, and carbohydrate contents (6). This study included 5 different strains: C57BL/6 (24 diets), BALB/c, C3H, FVB, and DBA/2 (12 diets) exposed to the various diets from age 16 weeks onward for 10 weeks. We previously investigated the impact of these diets on mean body weight, food intake, hypothalamic gene expression (7), glucose tolerance ability (26, 31), and senescent cell populations in the liver (32). In the present study, we analyzed the associations between dietary macronutrient levels and individual variations of several metabolic phenotypes in different strains.

## Experimental Procedures

### Mice and Experimental Diet

Data in the current article pertain to mice involved in a large dietary manipulation experiment, some aspects of which have already been published. These previous publications have included patterns of body weight, adiposity, hypothalamic gene expression (7, 31), and glucose homeostasis (29). All procedures in this study were reviewed and approved by the Institutional Review Board, Institute of Genetics and Developmental Biology, Chinese Academy of Sciences. C57BL/6N, DBA/2, BALB/c, FVB, and C3H mouse strains were used. C57BL/6N mouse strain was fed with 4 different diet series (series 1, 2, 3, and 4), and DBA/2, BALB/c, FVB, and C3H mouse strains were treated with 2 diet series (series 1 and 3) (Table 1). In the first two diet series (series 1: D14071601–D14071606, series 2: D14071607–D14071612), we fixed the level of fat 60 or 20% by energy and varied the protein content from 5 to 30% (5, 10, 15, 20, 25, and 30%, respectively) by energy. In the second two series of diets (series 3: D14071613–D14071618 and series 4: D14071619–D14071624), we fixed the level of protein at 10% (series 3) (10, 30, 40, 50, 70, and 80%, respectively) or 25% (series 4) (8.3, 25, 33.3, 41.7, 58.3, and 66.6%, respectively) by energy and varied the fat content from 8.3 to 80% by energy. For full details of the diets, refer to Supplementary Tables S4–S8. During the whole experimental period, mice were singly housed under controlled 22–24°C temperature and 12:12 light-dark cycle conditions. Mice were killed by rising concentrations of CO_2_ for the collection of tissues and serum, which were quickly snap-frozen in liquid nitrogen and then stored in an −80°C freezer until analysis. More information about procedures and experimental designs can be found in Table 1 and in our previous articles [Table 1, (7, 31)].

**Table 1:** Summary of experiments performed.

**Experiments**	**Design**
Experiment 1: manipulation of dietary protein levels under fixed fat contents	a. Two series of 6 diets with varying protein level (5, 10, 15, 20, 25, and 30%) b. Series 1 had 60% fat and series 2 had 20% fat content c. C57BL/6 mice exposed to all 12 diets d. BALB/c, C3H, DBA/2, FVB mouse strains exposed to 6 diets with high fat (series 1)
Experiment 2: manipulation of dietary fat levels under fixed protein contents	a. Two series of 6 diets with varying fat level (10% to 80% and 8.3% to 66.6%) b. Series 3 had 10% protein and series 4 had 25% protein content c. C57BL/6 mice exposed to all 12 diets d. BALB/c, C3H, DBA/2, and FVB mouse strains exposed to 6 diets with 10% protein (series 3)

### Statistical Analysis

We used the coefficient of variation (CV) to express the variation in average body weight, food intake, and glucose tolerance ability of mice exposed to different diets with varying protein, fat, and carbohydrate contents. CV was calculated by dividing SD by the mean values of the dataset of body weight and food intake of each diet group. To reduce the skew, we used log transformed data. Multiple regression analysis with logged dietary protein, fat, and carbohydrate as predictors logged CV of body weight, food intake, fasting glucose level, and AUC as responses was used to analyze the relationships between predictor and responses. We also analyzed variation data using generalized linear modeling (GLM) with CV as the dependent variable, strain as a factor, and dietary levels of fat, protein, and carbohydrate contents as the covariates.

## Results

### Variations in Body Weight and Food Intake Were Significantly Related to the Dietary Fat Content

**Figure 1:**
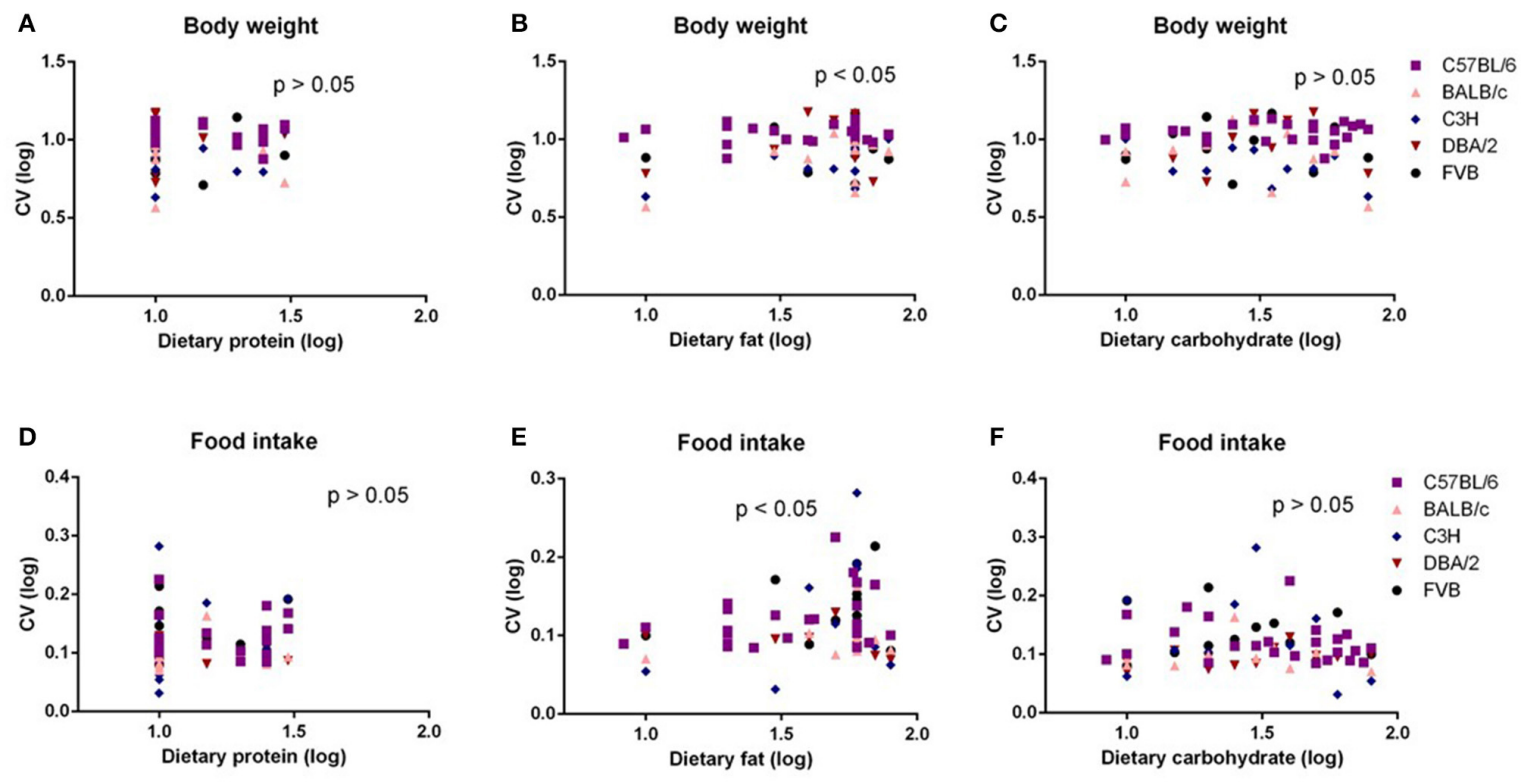
The diagram showing the relationship between dietary macronutrient content and the coefficient of variation (CV) of body weight and food intake. The correlations between logged dietary fat, protein, and carbohydrate contents and **(A–C)** logged CV of body weight and **(D–F)** food intake of the last week (10th week) at different diet treatment periods, respectively (*n* = 7–21).

In our previous study (6), we found no significant correlation between energy intake or body composition when the dietary protein content varied between 5 and 30%. However, an increased dietary fat content (8.3–80% fat) was associated with an elevated energy intake and adiposity up to around 50–60% fat; thereafter, there was a slight decrease. These effects were replicated at different levels in all the five mouse strains. To investigate the relationships between dietary macronutrient levels and variation in physiological traits, we used multiple regression analysis including data across all the diets with the percent dietary protein, fat, and carbohydrate contents as predictors and the CV of body weight and food intake during the last week (10th week) of dietary exposure as the dependent variables. Each strain by the diet combination generated a unique data point for the analysis. We found the CV of body weight and food intake were both significantly related to dietary fat levels (*p* = 0.036, *R*^2^ (unadj) = 0.07, *R*^2^ (adj) = 0.07, β = 0.251) and (*p* = 0.024, *R*^2^ (unadj) = 0.08, *R*^2^ (adj) = 0.038, β = 0.415, respectively) (Figures 1B,E), whereas there were no significant relationships between CV of body weight or food intake and dietary protein or carbohydrate content (*p* > 0.05) (Figures 1A,C,D,F). We also analyzed variation data using GLM with CV as the dependent variable, strain as a factor, and dietary levels of fat, protein, and carbohydrate contents as the covariates. In this analysis, we also found that CV of body weight and food intake was significantly affected by the dietary fat content (*p* = 0.008 and 0.019, respectively) but not the protein or carbohydrate content (*p* > 0.05). Furthermore, there was a significant effect of different strains on the CV of body weight and food intake (*p* = 0.014 and 0.025, respectively).

**Figure 2:**
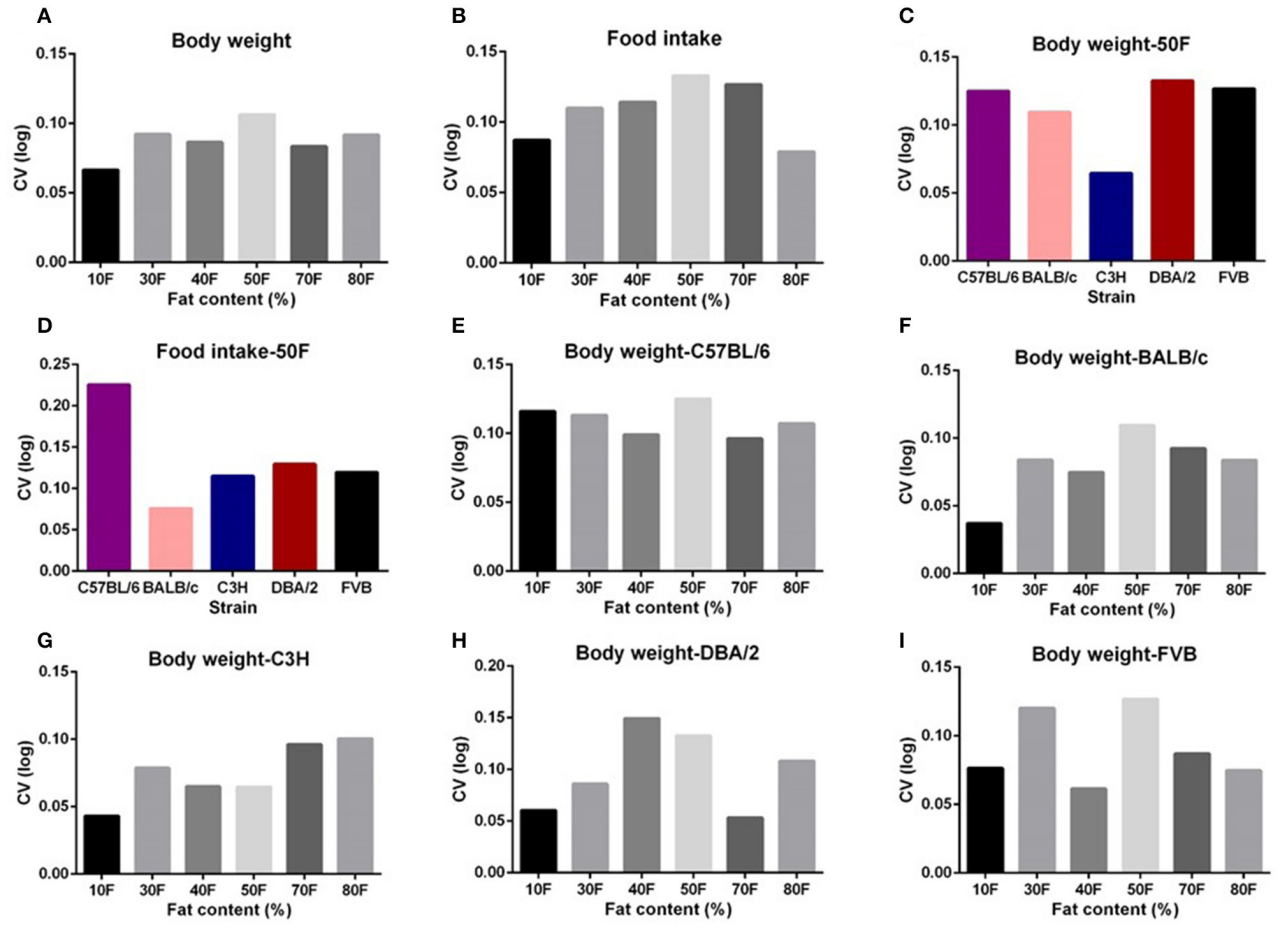
The coefficient of variation (CV) of body weight and food intake of different strains and different diet treatment groups. The CV of body weight and food intake of **(A,B)** graded levels of fat content diet treatment group, **(C,D)** different strains (*n* = 7–21). **(E–I)** Variations in body weight gain (CV) of five different strains (C57BL/6, BALB/c, C3H, DBA/2, and FVB) when fed with 10, 30, 40, 50, 70, and 80% content fat diets, respectively (*n* = 7–21).

The highest average variation (CV) of body weight and food intake across all the five strains was observed at the 50% dietary fat level (CV = 10.6 and 13.3%, respectively) (Figures 2A,B). When we compared the variation in body weight and food intake of each strain when exposed to 50% fat, the DBA/2 and C57BL/6 strains had the highest variation (CV = 9.8 and 14.1%, respectively) (Figures 2C,D). However, the highest variation in different strains across different fat content diets appeared at different fat levels. The C57BL/6, BALB/c, and FVB strains had the highest variation at 50% fat (12.5, 10.9, and 12.7%, respectively), whereas for C3H mice, variation at 80% dietary fat was highest (10.1%) and for DBA/2 mice variation was highest at 40% dietary fat (14.9%) (Figures 2E–I).

To explore whether the variation in food intake is the cause of the variation in body weight, we also used regression analysis between these two variables. We found no significant relationship between variations in body weight and food intake (*p* > 0.05). We also used multiple regression analysis with the percent dietary protein, fat, and carbohydrate contents and variation in food intake as predictors and variation in body weight as the dependent variables, we also did not find any significant relationship between variation in food intake and body weight in this analysis (*p* > 0.05).

### Variations in Fat Mass and Lean Mass Were Not Significantly Correlated With the Dietary Macronutrient Content

**Figure 3:**
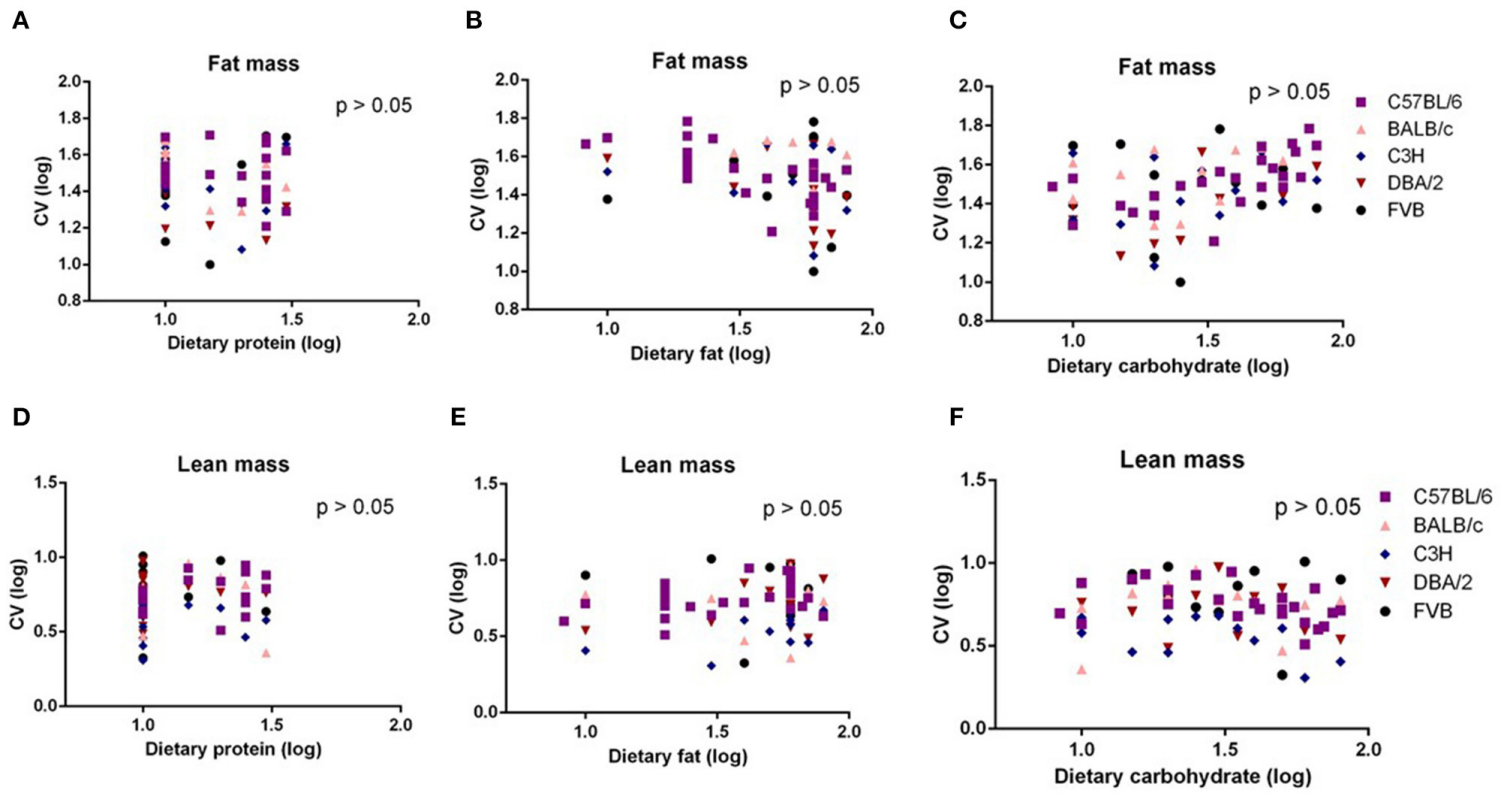
The diagram showing the relationship between dietary macronutrient content and the coefficient of variation (CV) of fat mass and lean mass. The correlations between logged dietary fat, protein, and carbohydrate contents and **(A–C)** logged CV of fat mass and **(D–F)** lean mass of the last week (10th week) at different diet treatment periods, respectively (*n* = 7–21).

Body composition analysis of our previous large diet manipulation studies indicated that body fat mass and lean mass changes were the same as the changes in body weight. That is, the increasing dietary fat content up to 60% fat caused increased fat and lean contents; however, further increase in the fat content led to a slight decrease in the fat mass. To investigate whether the significant dietary fat effect on the variation in body weight was the result of the variation changes in the body fat mass or lean mass, we also used multiple regression between variation (CV) in body fat mass or body lean mass and dietary protein, fat, or carbohydrate content. We found there were no significant associations between any of the dietary macronutrients and variation in body fat mass or lean mass (*p* > 0.05) (Figures 3A–F).

### Variations in Body Weight and Food Intake Across Different Diet Treatment Periods

**Figure 4:**
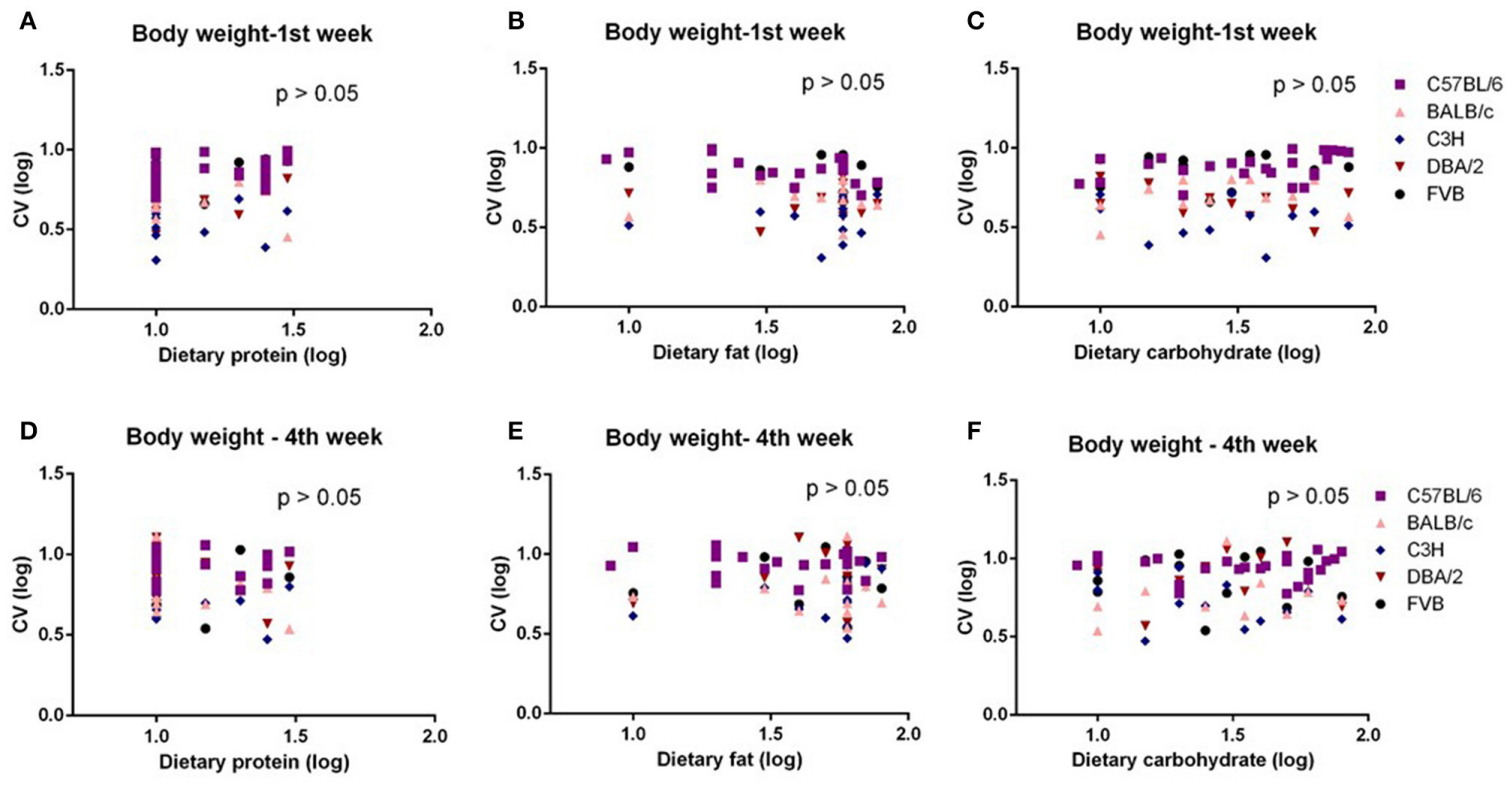
The diagram showing the relationship between dietary macronutrient content and coefficient of variation (CV) of body weight of the 1st week and 4th week after exposure to different diets. The correlations between logged dietary fat, protein, and carbohydrate contents and **(A–C)** logged CV of body weight of the 1st week and **(D–F)** 4th week at different diet treatment periods, respectively (*n* = 7–21).

**Figure 5:**
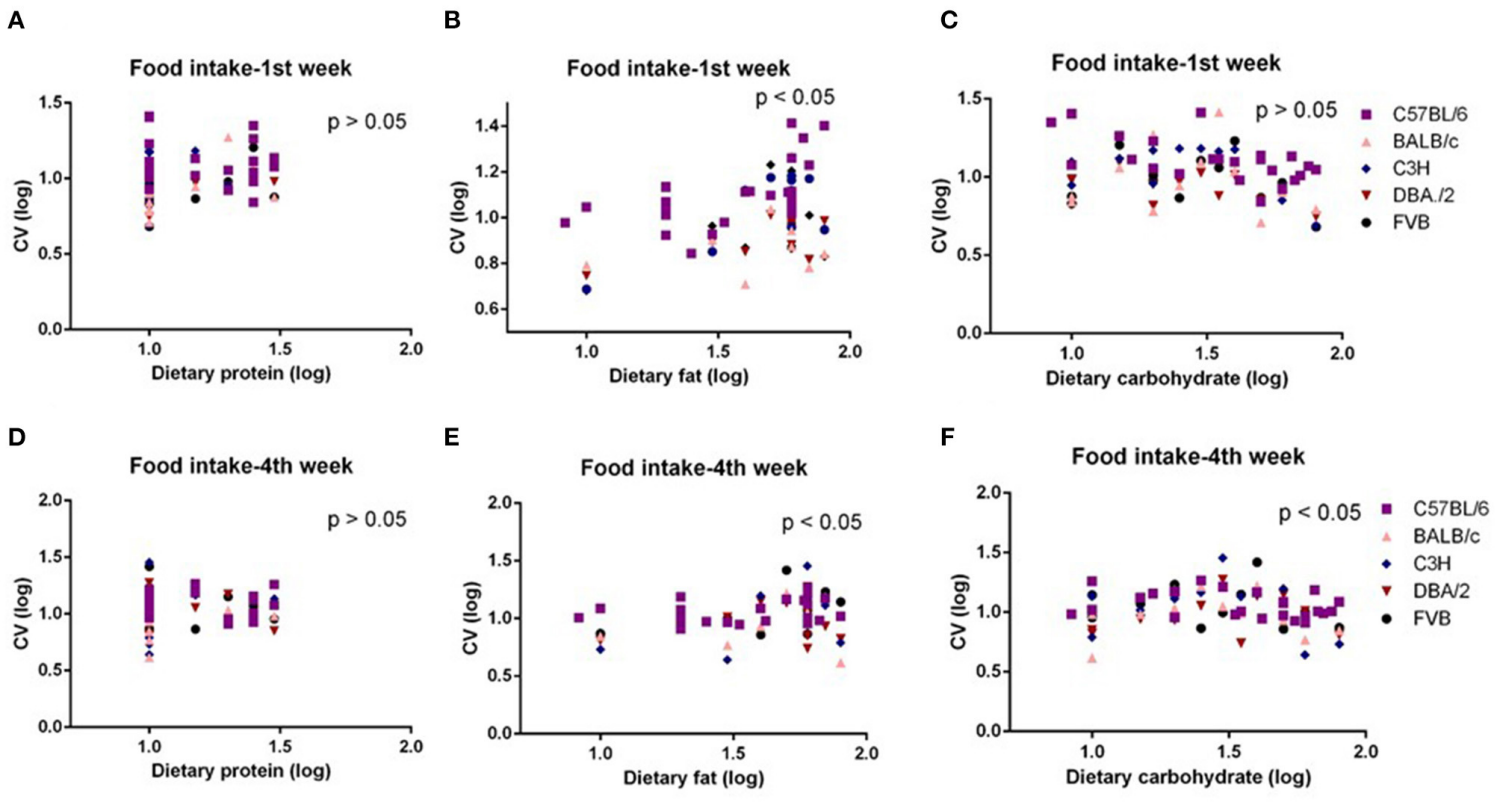
The diagram showing the relationship between dietary macronutrient content and coefficient of variation (CV) of food intake of the 1st week and 4th week after exposure to different diets. The correlations between logged dietary fat, protein, and carbohydrate contents and **(A–C)** logged CV of food intake of the 1st week and **(D–F)** 4th week at different diet treatment periods, respectively (*n* = 7–21).

The mice were treated with diets with varying content of protein, fat, and carbohydrate for 10 weeks (C57BL/6 mouse strain was treated for 12 weeks). The changing patterns of mean body weight and food intake with the alteration of dietary protein, fat, and carbohydrate contents were the same whether we used the average data over the entire 10 weeks or the average data of final weeks in all 5 strains. Therefore, we also used multiple regression to explore dietary impacts on variation in average body weight and food intake during weeks 1 and 4 of the dietary exposures. We found only the variation in body weight of 10 weeks was significantly correlated with dietary fat content (*p* = 0.0326, *R*^2^ (unadj) = 0.07, *R*^2^ (adj) = 0.07, β = 0.251) (Figure 1B). The variations in body weight after 1 and 4 weeks of dietary exposure had no significant associations with either dietary fat content or protein and carbohydrate contents (*p* > 0.05) (Figures 4A–F). However, the variation in food intake during the first week of diet exposure was significantly related to the dietary fat content (*p* = 0.014, *R*^2^ (unadj) = 0.16, *R*^2^ (adj) = 0.12, β = 0.358) (Figure 5B) but not protein and carbohydrate contents (*p* > 0.05) (Figures 5A,C). In week 4, there were significant associations between the variation in food intake and both dietary fat and carbohydrate contents of the diets (*p* < 0.001, *R*^2^ (unadj) = 0.18, *R*^2^ (adj) = 0.14, β = 0.516 for fat and *p* = 0.007, *R*^2^ (unadj) = 0.16, *R*^2^ (adj) = 0.12, β = 0.376 for carbohydrate, respectively) (Figures 5D–F).

### Variation in Glucose Tolerance Ability Was Not Related to the Dietary Macronutrients

**Figure 6:**
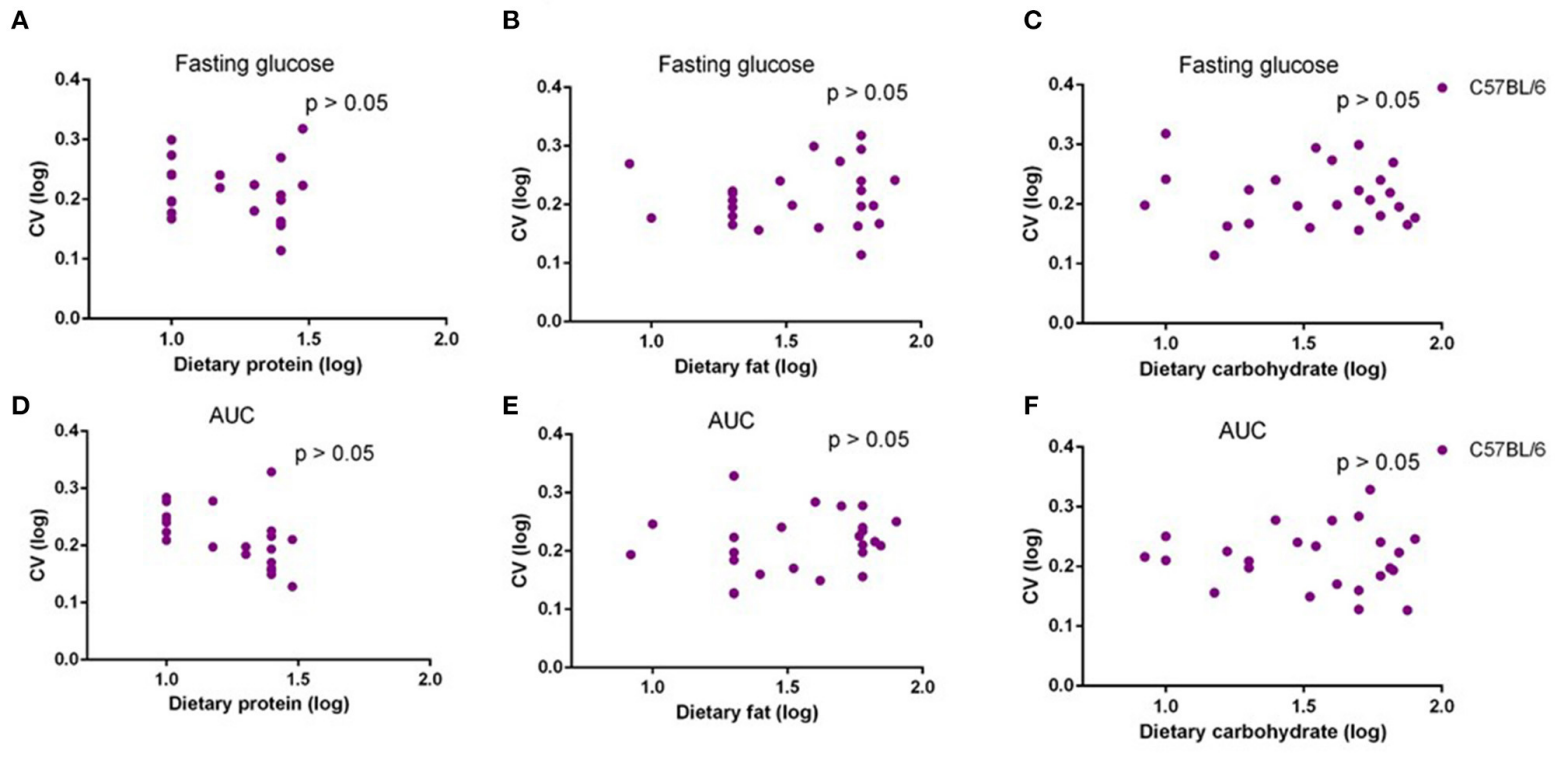
The diagram showing the relationship between dietary macronutrient content and coefficient of variation (CV) of fasting glucose and area under the curve (AUC) of C57BL/6 mice after exposure to different diets. The correlations between logged dietary fat, protein, and carbohydrate contents and **(A–C)** logged CV of fasting glucose and **(D–F)** AUC of last week at different diet treatment periods, respectively (*n* = 10–11).

In our previous study, we fed C57BL/6 mice with 29 different diets with variable macronutrients for 12 weeks, and an intraperitoneal glucose tolerance test (IPGTT) was used after 10 weeks. We found that the area under the glucose curve (AUC) was strongly associated with body fat mass, but once that effect was taken into account, AUC was not associated with different dietary macronutrients (29). In the present study, we also used correlations between the dietary protein, fat, and carbohydrate contents, and the CV in fasting glucose levels and AUC. There were no significant associations between CV of fasting glucose level or AUC and dietary protein, fat, or carbohydrate level (*p* > 0.05) (Figures 6A–F). Analysis of covariance (ANCOVA) with fat mass as a covariate (to remove the fat mass effect) also showed that CV of AUC was not significantly affected by dietary protein, fat, or carbohydrate content.

## Discussion

The C57BL/6 mice have been previously shown to display high variation in various metabolic traits when fed with high-fat diets (generally comprising 45–60% fat) (19, 20, 28, 33, 34). Consistent with these previous studies, we also found a high variation in body weight and food intake in C57BL/6 mice when fed with different macronutrient content diets, and that the highest variation in these traits occurred at the 50% dietary fat level. It has been indicated in a previous study that variations in body weight gain under a high-fat diet in male C57BL/6 mice were related to baseline fat mass, fat-free mass, and physical activity (19). A further study confirmed that the baseline fat mass and the change in energy intake on exposure to the new diet were predictors of body weight gain when fed with a high-fat diet in both male and female C57BL/6 mice (34). We have traced this baseline variation in body fatness and changes in energy intake to differentiate the nutritional environment experienced by young mice during lactation (15). Maternal milk production is constrained by the capacity to dissipate body heat (35); hence, as litter size increases, the pups must share a limited resource, and this means mice from larger litters wean with smaller body sizes and fatness, traits which persist into later life (15). Feeding the mother a high-fat diet can also affect milk production (36) and pup size/fatness at weaning.

As demonstrated here, the extent of variation among the individuals in both food intake and body weight was only related to differences in the dietary fat content. It is important to note that because we expressed the variation as a CV, this was not simply a reflection of the changing mean levels of body weight under different exposures, which were also highest under high-fat feeding. Mice not only became on average heavier when fed with high-fat diets but also became more variable in their body weights. In contrast when exposed to, e.g., low-protein or high-carbohydrate diets, they did not become more variable in their responses. This suggests that the early-life programming in lactation seems only to prime the individuals to respond differently to dietary fat, and not to the other macronutrients. The reasons for this difference are unclear at present. It is also unclear if the early-life priming by other macronutrients (e.g., low protein or high sugar) would generate similar later-life differences in variation between individuals in response to the same macronutrients.

A variety of mouse strains have been used to study metabolic disorders. Early studies investigated the strain differences in metabolic phenotypes like weight gain and insulin resistance (10, 37–39). For example, they found that C57BL/6, DBA/2, FVB, BALB/c, and 129X1 mice are all susceptible to diet-induced weight gain when fed with a high-fat diet, but BALB/c mice displayed unchanged glucose tolerance and insulin action compared to the other strains that showed impaired glucose tolerance after fed with a high-fat diet (39). However, there is controversy about whether those mouse strains were obesity-prone or obesity-resistant. For example, FVB and DBA/2 mouse strains have been described as both obesity-prone and obesity-resistant (13, 15), whereas few studies investigated the variations in metabolic traits of different strains and their relationship to the dietary macronutrient content. It was interesting that the variations in fat mass and lean mass were not significantly associated with the dietary fat content. Therefore, the variations in body weight gain with the increase of dietary fat content cannot be explained by the variations in fat mass or lean mass alone. There were also no significant relationships between variations in body weight and food intake, which means the higher variation in food intake is not the direct factor caused by the higher variation in body weight. Therefore, the variations in both energy intake and expenditure may cause higher variations in body weight.

We found previously that only an increased dietary fat content was associated with an elevated energy intake and adiposity, and this was related to the increased gene expression in 5-HT receptors, and the dopamine and opioid signaling pathways in the hypothalamus in C57BL/6 mice (7). Because variations in body weight and food intake were both increased with an elevation of dietary fat content, the potential mechanism creating this variation may be linked to differences in these hypothalamic pathway changes (opioid and dopamine) in these conditions. Supporting this, C57BL/6 mice displayed a significant food intake variance when exposed to a high-fat diet for 4 consecutive days, and mice displaying higher-fat intake showed an increased c-Fos expression in dopamine neurons in the ventral tegmental area (VTA) compared to lower-fat intake group (23). However, there is no mechanism study to prove causality in this association. Further studies are required to elucidate the potential mechanism in the hypothalamic signaling pathway that may underlie the variation in weight gain under high-fat diet conditions.

Increased adiposity is linked to the development of type 2 diabetes (25). In the present study, we found there were no significant associations between the variations in fasting glucose level and AUC of glucose tolerance test and any of the dietary macronutrient contents. However, several previous studies reported that elevated adiposity is not linked to metabolic dysfunction (26, 27). For example, the percent of the mice that became obese and diabetic after feeding C57BL/6 mice with a high-fat (72% fat) carbohydrate-free diet for 9 months (28) included 47% of mice that became obese and diabetic, 10% lean and diabetic, 10% lean and non-diabetic, and 30% showed intermediate phenotype (28). We analyzed the percent of the mice that were diabetic or not using the previously established criteria (27). We found that under both 50% fat (higher variation occurred) and 70% fat (the same fat level as the previous study), only 10% of mice became diabetic, 50% of mice remained nondiabetic, and 40% of mice displayed an intermediate phenotype. The difference between studies was similar because the diet exposure in our study was only 10 weeks compared to 9 months earlier, indicating that progression from obesity to diabetes is time-dependent. Furthermore, it has been shown that the variations in glucose tolerance and insulin resistance are also strain-dependent as they indicated that C57BL/6 mice are more insulin-sensitive than AKR mice (30).

In summary, we demonstrated that the variations in body weight and food intake were significantly increased in relation to the elevation of dietary fat level in all the five mouse strains but not in relation to changes in the level of dietary carbohydrates and protein. Since we previously traced this individual variability to individual differences in early-life nutrition, it is unclear why such early-life experience leads to such high variability only in response to high levels of dietary fat exposure. More study to understand the basis of the non-genetic variability in responses to diets is needed.

## Data Availability Statement

The raw data supporting the conclusions of this article will be made available by the authors, without undue reservation.

## Ethics Statement

The animal study was reviewed and approved by Institutional Review Board, Institute of Genetics and Developmental Biology, Chinese Academy of Sciences.

## Author Contributions

YW contributed to the data collection, analyzed the data and co-wrote the manuscript. JS directed the project, conceived and designed the experiments, contributed to the analysis, and co-wrote the manuscript. DY, BL, LL, GW, LW, ML, JL, SH, CN, XZ, and YX contributed to the data collection. All the authors approved the final version prior to submission for publication.

## Funding

This study was funded by the National Key R&D Program of China (2019YFA0801900) to JS and the Postdoctoral Innovation Fund (2021) to YW. The original diet exposure experiment was funded by the Chinese Academy of Sciences Strategic Program (XDB13030100). JS was also supported during this work by a PIFI professorial fellowship from CAS and a Wolfson merit award from the UK Royal Society.

## Conflict of Interest

The authors declare that the research was conducted in the absence of any commercial or financial relationships that could be construed as a potential conflict of interest.

## Publisher's Note

All claims expressed in this article are solely those of the authors and do not necessarily represent those of their affiliated organizations, or those of the publisher, the editors and the reviewers. Any product that may be evaluated in this article, or claim that may be made by its manufacturer, is not guaranteed or endorsed by the publisher.
