# Corrigendum: Increased variation in body weight and food intake is related to increased dietary fat but not increased carbohydrate or protein in mice

**DOI:** 10.3389/fnut.2022.1049766

**Published:** 2022-10-21

**Authors:** Yingga Wu, Sumei Hu, Dengbao Yang, Li Li, Baoguo Li, Lu Wang, Min Li, Guanlin Wang, Jianbo Li, Yanchao Xu, Xueying Zhang, Chaoqun Niu, John R. Speakman

**Affiliations:** ^1^State Key Laboratory of Molecular Developmental Biology, Institute of Genetics and Developmental Biology, Chinese Academy of Sciences, Beijing, China; ^2^University of Chinese Academy of Sciences, Beijing, China; ^3^Institute of Biological and Environmental Sciences, University of Aberdeen, Aberdeen, United Kingdom; ^4^Beijing Advanced Innovation Center for Food Nutrition and Human Health, Beijing Engineering and Technology Research Center of Food Additives, National Soybean Processing Industry Technology Innovation Center, Beijing Technology and Business University, Beijing, China; ^5^Shenzhen Key Laboratory of Metabolic Health, Center for Energy Metabolism and Reproduction, Shenzhen Institutes of Advanced Technology, Chinese Academy of Sciences, Shenzhen, China; ^6^University of Dali, Dali, China; ^7^CAS Center of Excellence in Animal Evolution and Genetics, Kunming, China

**Keywords:** protein, fat, carbohydrate, mice, strain, variation

In the published article, there was an error in affiliation [4]. Instead of “Beijing Advanced Innovation Center for Food Nutrition and Human Health, Beijing Engineering and Technology Research Center of Food Additives, National Soybean Processing Industry Technology Innovation Center, Beijing, China,” it should be “Beijing Advanced Innovation Center for Food Nutrition and Human Health, Beijing Engineering and Technology Research Center of Food Additives, National Soybean Processing Industry Technology Innovation Center, Beijing Technology and Business University, Beijing, China.”

The authors apologize for this error and state that this does not change the scientific conclusions of the article in any way. The original article has been updated.

## Publisher's note

All claims expressed in this article are solely those of the authors and do not necessarily represent those of their affiliated organizations, or those of the publisher, the editors and the reviewers. Any product that may be evaluated in this article, or claim that may be made by its manufacturer, is not guaranteed or endorsed by the publisher.

