# Supplementary material for: Increased Variation in Body Weight and Food Intake Is Related to Increased Dietary Fat but Not Increased Carbohydrate or Protein in Mice
Source: Front Nutr. 2022 Mar 8;9:835536. doi: 10.3389/fnut.2022.835536 (PMC8963818; doi:10.3389/fnut.2022.835536)
Supplement: Supplementary file 1 [file Data_Sheet_1.pdf]

Table S1. The physiological parameters of mice used in the analysis of multiple regression between dietary protein content and CV of body weight.

Group	Strain	Fat%	Protein%	Carbo%	Mean BW(g)	CV%	Number of mice
1	C57BL/6	60	5	35	38.84	12.1	21
2	C57BL/6	60	10	30	43.81	11.04	21
3	C57BL/6	60	15	25	44.57	10.48	21
4	C57BL/6	60	20	20	45.54	7.47	21
5	C57BL/6	60	25	15	45.13	9.68	20
6	C57BL/6	60	30	10	45.27	10.78	21
7	C57BL/6	20	5	75	31.05	12.23	21
8	C57BL/6	20	10	70	35.38	9.77	21
9	C57BL/6	20	15	65	35.41	12.52	21
10	C57BL/6	20	20	60	36.3	8.79	18
11	C57BL/6	20	25	55	35.7	7.53	20
12	C57BL/6	20	30	50	34.06	10.66	20
13	BALB/c	60	5	35	29.55	4.57	8
14	BALB/c	60	10	30	35.34	12.93	8
15	BALB/c	60	15	25	34.17	13.44	8
16	BALB/c	60	20	20	37.42	9.73	8
17	BALB/c	60	25	15	34.42	8.57	8
18	BALB/c	60	30	10	31.14	5.34	8
19	C3H	60	5	35	32.34	4.82	8
20	C3H	60	10	30	34.46	8.58	8
21	C3H	60	15	25	37.75	8.84	8
22	C3H	60	20	20	40.25	6.28	8
23	C3H	60	25	15	38.8	6.24	8
24	C3H	60	30	10	37.56	11.45	8
25	DBA/2	60	5	35	36.38	8.81	8
26	DBA/2	60	10	30	37.88	14.6	8
27	DBA/2	60	15	25	38.42	10.27	8
28	DBA/2	60	20	20	39.26	9.2	8
29	DBA/2	60	25	15	38.2	7.54	8
30	DBA/2	60	30	10	40.3	10.86	8
31	FVB	60	5	35	36.38	14.71	8
32	FVB	60	10	30	37.71	9.91	8
33	FVB	60	15	25	37.26	5.14	8
34	FVB	60	20	20	37.78	14	8
35	FVB	60	25	15	35.08	10.92	7
36	FVB	60	30	10	35.43	7.98	7

Table S2. The physiological parameters of mice used in the analysis of multiple regression between dietary fat content and CV of body weight.

Group	Strain	Fat%	Protein%	Carbo%	Mean BW(g)	CV%	Number of mice
1	C57BL/6	10	10	80	34.44	11.24	20
2	C57BL/6	30	10	60	39.21	9.2	20
3	C57BL/6	40	10	50	40.74	8.18	19
4	C57BL/6	50	10	40	43.38	9.82	19
5	C57BL/6	70	10	20	40.13	8.81	20
6	C57BL/6	80	10	10	38.8	9.21	20
7	C57BL/6	8.3	25	66.7	33.32	9.16	20
8	C57BL/6	25	25	50	35.25	10.86	20
9	C57BL/6	33.3	25	41.7	39.3	8.92	20
10	C57BL/6	41.7	25	33.3	45.02	9.65	19
11	C57BL/6	58.3	25	16.7	46.71	10.54	20
12	C57BL/6	66.6	25	8.4	39.73	9.71	20
13	BALB/c	10	10	80	28.25	3.69	8
14	BALB/c	30	10	60	30.56	8.39	8
15	BALB/c	40	10	50	32.87	7.48	8
16	BALB/c	50	10	40	35.44	10.95	8
17	BALB/c	70	10	20	33.98	9.24	8
18	BALB/c	80	10	10	33.28	8.38	8
19	C3H	10	10	80	29.35	4.3	8
20	C3H	30	10	60	34.84	7.87	8
21	C3H	40	10	50	35.52	6.49	8
22	C3H	50	10	40	37.2	6.46	8
23	C3H	70	10	20	38.96	9.62	8
24	C3H	80	10	10	38.22	10.06	8
25	DBA/2	10	10	80	27	6.03	8
26	DBA/2	30	10	60	31.28	8.6	8
27	DBA/2	40	10	50	35.06	14.93	8
28	DBA/2	50	10	40	38.6	13.26	8
29	DBA/2	70	10	20	38.76	5.33	8
30	DBA/2	80	10	10	39.1	10.8	8
31	FVB	10	10	80	31.28	7.64	8
32	FVB	30	10	60	32.35	12.02	8
33	FVB	40	10	50	32.65	6.14	8
34	FVB	50	10	40	37.27	12.68	9
35	FVB	70	10	20	39.83	8.71	7
36	FVB	80	10	10	39.8	7.47	8

Table S3. The physiological parameters of mice used in the analysis of multiple regression between dietary protein content and CV of food intake.

Group	Strain	Fat%	Protein%	Carbo%	Mean FI(g)	CV%	Number of mice
1	C57BL/6	60	5	35	3.82	10.3	21
2	C57BL/6	60	10	30	3.22	11.48	21
3	C57BL/6	60	15	25	3.24	11.38	21
4	C57BL/6	60	20	20	3.17	8.5	21
5	C57BL/6	60	25	15	3.2	13.8	20
6	C57BL/6	60	30	10	3.34	16.8	21
7	C57BL/6	20	5	75	3.98	8.61	21
8	C57BL/6	20	10	70	3.5	10.63	21
9	C57BL/6	20	15	65	3.61	13.37	21
10	C57BL/6	20	20	60	3.5	10.32	18
11	C57BL/6	20	25	55	3.46	9.02	20
12	C57BL/6	20	30	50	3.41	14.12	20
13	BALB/c	60	5	35	4.27	11.14	8
14	BALB/c	60	10	30	3.51	9.36	8
15	BALB/c	60	15	25	3.62	16.34	8
16	BALB/c	60	20	20	3.35	9.89	8
17	BALB/c	60	25	15	3.27	8.05	8
18	BALB/c	60	30	10	2.97	9.32	8
19	C3H	60	5	35	4.87	10.67	8
20	C3H	60	10	30	4.55	28.21	8
21	C3H	60	15	25	4.49	18.56	8
22	C3H	60	20	20	4.6	10.42	8
23	C3H	60	25	15	4.58	10.76	8
24	C3H	60	30	10	4.21	19.23	8
25	DBA/2	60	5	35	3.87	11.12	8
26	DBA/2	60	10	30	3.42	8.4	8
27	DBA/2	60	15	25	3.49	8.1	8
28	DBA/2	60	20	20	3.5	9.4	8
29	DBA/2	60	25	15	3.53	10.68	8
30	DBA/2	60	30	10	3.61	8.69	8
31	FVB	60	5	35	4.27	15.32	8
32	FVB	60	10	30	3.62	14.64	8
33	FVB	60	15	25	3.54	12.56	8
34	FVB	60	20	20	3.38	11.47	8
35	FVB	60	25	15	3.49	10.33	7
36	FVB	60	30	10	3.48	19.12	7

Table S4. The physiological parameters of mice used in the analysis of multiple regression between dietary fat content and CV of food intake.

Group	Strain	Fat%	Protein%	Carbo%	Mean FI(g)	CV%	Number of mice
1	C57BL/6	10	10	80	3.67	11.04	20
2	C57BL/6	30	10	60	3.58	12.62	20
3	C57BL/6	40	10	50	3.56	12.07	19
4	C57BL/6	50	10	40	3.82	22.54	19
5	C57BL/6	70	10	20	3.05	16.48	20
6	C57BL/6	80	10	10	2.59	10.07	20
7	C57BL/6	8.3	25	66.7	3.51	8.96	20
8	C57BL/6	25	25	50	3.35	8.43	20
9	C57BL/6	33.3	25	41.7	3.2	9.71	20
10	C57BL/6	41.7	25	33.3	3.31	12.12	19
11	C57BL/6	58.3	25	16.7	3.25	18.06	20
12	C57BL/6	66.6	25	8.4	2.96	9.1	20
13	BALB/c	10	10	80	4.03	7.04	8
14	BALB/c	30	10	60	3.69	12.55	8
15	BALB/c	40	10	50	3.89	10.32	8
16	BALB/c	50	10	40	4.15	7.58	8
17	BALB/c	70	10	20	3.15	9.51	8
18	BALB/c	80	10	10	2.82	8.2	8
19	C3H	10	10	80	4.95	5.44	8
20	C3H	30	10	60	4.51	3.15	8
21	C3H	40	10	50	4.99	16.12	8
22	C3H	50	10	40	5.27	11.5	8
23	C3H	70	10	20	4.	8.56	8
24	C3H	80	10	10	3.17	6.25	8
25	DBA/2	10	10	80	4.21	10.12	8
26	DBA/2	30	10	60	3.72	9.53	8
27	DBA/2	40	10	50	3.76	9.77	8
28	DBA/2	50	10	40	3.74	12.94	8
29	DBA/2	70	10	20	3.23	7.44	8
30	DBA/2	80	10	10	3.17	6.92	8
31	FVB	10	10	80	4.21	10.02	8
32	FVB	30	10	60	3.96	17.14	8
33	FVB	40	10	50	3.72	8.88	8
34	FVB	50	10	40	3.58	11.96	9
35	FVB	70	10	20	3.5	21.42	7
36	FVB	80	10	10	3.05	8.08	8

Table S5. 60%fat and different protein content diet composition table.

**Product #**	**5 kcal% Protein**		**10 kcal % Protein**		**15 kcal % Protein**		**20 kcal % Protein**		**25 kcal % Protein**		**30 kcal % Protein**	
	**gm%**	**kcal%**	**gm%**	**kcal%**	**gm%**	**kcal%**	**kcal%**	**kcal%**	**gm%**	**kcal%**	**gm%**	**kcal%**
**Protein**	**7**	**5**	**13**	**10**	**20**	**15**	**26**	**20**	**33**	**25**	**39**	**30**
Carbohydrate	46	35	39	30	33	25	26	20	20	15	13	10
**Fat**	**35**	**60**	**35**	**60**	**35**	**60**	**35**	**60**	**35**	**60**	**35**	**60**
Total		100		100		100		100		100		100
kcal/gm	5.2		5.2		5.2		5.2		5.2		5.1	
**Ingredient**	**gm**	**kcal**	**gm**	**kcal**	**gm**	**kcal**	**gm**	**kcal**	**gm**	**kcal**	**gm**	**kcal**
Casein	50	200	100	400	150	600	200	800	250	1000	300	1200
L-Cystine	0.75	3	1.5	6	2.25	9	3	12	3.75	15	4.5	18
Corn Starch	145	580	114.3	457	68.6	274	17.8	71	0	0	0	0
Maltodextrin 10	150	600	130	520	125	500	125	500	92	368	41.2	165
Sucrose	51	204	51	204	51	204	51	204	51	204	51	204
Cellulose, BW200	50	0	50	0	50	0	50	0	50	0	50	0
Cocoa Butter	75	675	75	675	75	675	75	675	75	675	75	675
Coconut Oil	5	45	5	45	5	45	5	45	5	45	5	45
Menhaden Oil	5	45	5	45	5	45	5	45	5	45	5	45
Palm Oil	165	1485	165	1485	165	1485	165	1485	165	1485	165	1485
Safflower Oil	20	180	20	180	20	180	20	180	20	180	20	180
Mineral Mix, S10026	10	0	10	0	10	0	10	0	10	0	10	0
DiCalcium Phosphate	13	0	13	0	13	0	13	0	13	0	13	0
Calcium Carbonate	5.5	0	5.5	0	5.5	0	5.5	0	5.5	0	5.5	0
Potassium Citrate, 1 H2O	16.5	0	16.5	0	16.5	0	16.5	0	16.5	0	16.5	0
Vitamin Mix, V10001	10	40	10	40	10	40	10	40	10	40	10	40
Choline Bitartrate	2	0	2	0	2	0	2	0	2	0	2	0
FD&C Yellow Dye #5	0	0	0	0	0	0	0	0	0	0	0	0
FD&C Red Dye #40	0.0125	0	0.05	0	0.075	0	0.125	0	0.175	0	0.2	0
FD&C Blue Dye #1	0	0	0	0	0	0	0	0	0	0	0	0
**Total**	**773.763**	**4057**	**773.85**	**4057**	**773.925**	**4057**	**773.925**	**4057**	**773.925**	**4057**	**773.925**	**4057**

Table S6. 20%fat and different protein content diet composition table.

Product #	5 kcal% Protein		10 kcal % Protein		15 kcal % Protein		20 kcal % Protein		25 kcal % Protein		30 kcal % Protein	
	**gm%**	**kcal%**	**gm%**	**kcal%**	**gm%**	**kcal%**	**kcal%**	**kcal%**	**gm%**	**kcal%**	**gm%**	**kcal%**
**Protein**	**5**	**5**	**10**	**10**	**15**	**15**	**20**	**20**	**25**	**25**	**30**	**30**
Carbohydrate	76	75	71	70	66	65	61	60	56	55	51	50
**Fat**	**9**	**20**	**9**	**20**	**9**	**20**	**9**	**20**	**9**	**20**	**9**	**20**
Total		100		100		100		100		100		100
kcal/gm	4.1		4.1		4.1		4.1		4.1		4.1	
**Ingredient**	**gm**	**kcal**	**gm**	**kcal**	**gm**	**kcal**	**gm**	**kcal**	**gm**	**kcal**	**gm**	**kcal**
Casein	50	200	100	400	150	600	200	800	250	1000	300	1200
L-Cystine	0.75	3	1.5	6	2.25	9	3	12	3.75	15	4.5	18
Corn Starch	600	2400	549.3	2197	498.5	1994	447.7	1791	397	1588	346.2	1385
Maltodextrin 10	100	400	100	400	100	400	100	400	100	400	100	400
Sucrose	51	204	51	204	51	204	51	204	51	204	51	204
Cellulose, BW200	50	0	50	0	50	0	50	0	50	0	50	0
Cocoa Butter	25	225	25	225	25	225	25	225	25	225	25	225
Coconut Oil	1.7	15	1.7	15	1.7	15	1.7	15	1.7	15	1.7	15
Menhaden Oil	1.7	15	1.7	15	1.7	15	1.7	15	1.7	15	1.7	15
Palm Oil	55	495	55	495	55	495	55	495	55	495	55	495
Safflower Oil	6.6	59	6.6	59	6.6	59	6.6	59	6.6	59	6.6	59
Mineral Mix, S10026	10	0	10	0	10	0	10	0	10	0	10	0
DiCalcium Phosphate	13	0	13	0	13	0	13	0	13	0	13	0
Calcium Carbonate	5.5	0	5.5	0	5.5	0	5.5	0	5.5	0	5.5	0
Potassium Citrate, 1 H2O	16.5	0	16.5	0	16.5	0	16.5	0	16.5	0	16.5	0
Vitamin Mix, V10001	10	40	10	40	10	40	10	40	10	40	10	40
Choline Bitartrate	2	0	2	0	2	0	2	0	2	0	2	0
FD&C Yellow Dye #5	0.0063	0	0.025	0	0.0375	0	0.0625	0	0.0875	0	0.1	0
FD&C Red Dye #40	0.0063	0	0.025	0	0.0375	0	0.0625	0	0.0875	0	0.1	0
FD&C Blue Dye #1	0	0	0	0	0	0	0	0	0	0	0	0
**Total**	**998.76**	**4057**	**998.85**	**4057**	**998.83**	**4057**	**998.83**	**4057**	**998.83**	**4057**	**998.9**	**4057**

Table S7. 10% protein and different fat content diet composition table.

Product #	10 kcal% Fat		30 kcal% Fat		40 kcal% Fat		50 kcal% Fat		70 kcal% Fat		80 kcal% Fat	
	gm%	kcal%	gm%	kcal%	gm%	kcal%	kcal%	kcal%	gm%	kcal%	gm%	kcal%
**Protein**	**10**	**10**	**11**	**10**	**11**	**10**	**12**	**10**	**14**	**10**	**15**	**10**
Carbohydrate	77	80	65	60	57	50	49	40	28	20	15	10
**Fat**	**4**	**10**	**14**	**30**	**20**	**40**	**27**	**50**	**44**	**70**	**54**	**80**
Total		100		100		100		100		100		100
kcal/gm	3.8		4.3		4.6		4.9		5.7		6.1	
**Ingredient**	**gm**	**kcal**	**gm**	**kcal**	**gm**	**kcal**	**gm**	**kcal**	**gm**	**kcal**	**gm**	**kcal**
Casein	100	400	100	400	100	400	100	400	100	400	100	400
L-Cystine	1.5	6	1.5	6	1.5	6	1.5	6	1.5	6	1.5	6
Corn Starch	650.6	2602	448	1792	347	1388	245.5	982	43.2	173	0	0
Maltodextrin 10	100	400	100	400	100	400	100	400	100	400	41.4	166
Sucrose	51	204	51	204	51	204	51	204	51	204	51	204
Cellulose, BW200	50	0	50	0	50	0	50	0	50	0	50	0
Cocoa Butter	12.5	113	37.5	338	50	450	62.5	563	87.5	788	100	900
Coconut Oil	0.85	8	2.5	23	3.3	30	4.2	38	5.8	52	6.7	60
Menhaden Oil	0.85	8	2.5	23	3.3	30	4.2	38	5.8	52	6.7	60
Palm Oil	27.5	248	82.5	743	110	990	137.5	1238	192.5	1733	220	1980
Safflower Oil	3.3	30	10	90	13.3	120	16.6	149	23.3	210	26.7	240
Mineral Mix, S10026	10	0	10	0	10	0	10	0	10	0	10	0
DiCalcium Phosphate	13	0	13	0	13	0	13	0	13	0	13	0
Calcium Carbonate	5.5	0	5.5	0	5.5	0	5.5	0	5.5	0	5.5	0
Potassium Citrate, 1 H2O	16.5	0	16.5	0	16.5	0	16.5	0	16.5	0	16.5	0
Vitamin Mix, V10001	10	40	10	40	10	40	10	40	10	40	10	40
Choline Bitartrate	2	0	2	0	2	0	2	0	2	0	2	0
FD&C Yellow Dye #5	0.0063	0	0.025	0	0.0375	0	0.0625	0	0.0875	0	0.125	0
FD&C Red Dye #40	0	0	0	0	0	0	0	0	0	0	0	0
FD&C Blue Dye #1	0.0063	0	0.025	0	0.0375	0	0.0625	0	0.0875	0	0.125	0
**Total**	**1055**	**4057**	**942.55**	**4057**	**886.48**	**4057**	**830.13**	**4057**	**717.78**	**4057**	**661.25**	**4057**

Table S8. 25% protein and different fat content diet composition table.

**Product #**	**8.3 kcal% Fat**		**25 kcal% Fat**		**33.3 kcal% Fat**		**41.7 kcal% Fat**		**58.3 kcal% Fat**		**66.6 kcal% Fat**	
	**gm%**	**kcal%**	**gm%**	**kcal%**	**gm%**	**kcal%**	**kcal%**	**kcal%**	**gm%**	**kcal%**	**gm%**	**kcal%**
**Protein**	**24**	**25**	**26**	**25**	**27**	**25**	**29**	**25**	**32**	**25**	**34**	**25**
Carbohydrate	64	67	52	50	46	42	39	33	22	17	12	8
**Fat**	**4**	**8.3**	**12**	**25**	**16**	**33.3**	**21**	**41.7**	**34**	**58.3**	**41**	**66.6**
Total		100		100		100		100		100		100
kcal/gm	3.8		4.2		4.4		4.6		5.2		6.1	
**Ingredient**	**gm**	**kcal**	**gm**	**kcal**	**gm**	**kcal**	**gm**	**kcal**	**gm**	**kcal**	**gm**	**kcal**
Casein	250	1000	250	1000	250	1000	250	1000	250	1000	250	1000
L-Cystine	3.75	15	3.75	15	3.75	15	3.75	15	3.75	15	3.75	15
Corn Starch	515.5	2062	346.5	1386	262	1048	176.5	706	8	32	0	0
Maltodextrin 10	100	400	100	400	100	400	100	400	100	400	24	96
Sucrose	51	204	51	204	51	204	51	204	51	204	51	204
Cellulose, BW200	50	0	50	0	50	0	50	0	50	0	50	0
Cocoa Butter	10.37	93	31.25	281	41.67	375	52.22	470	73.03	657	83.39	751
Coconut Oil	0.69	6	2.08	19	2.78	25	3.48	31	4.87	44	5.56	50
Menhaden Oil	0.69	6	2.08	19	2.78	25	3.48	31	4.87	44	5.56	50
Palm Oil	22.8	205	68.75	619	91.67	825	114.89	1034	160.66	1446	183.45	1651
Safflower Oil	2.77	25	8.33	75	11.1	100	13.92	125	19.47	175	22.24	200
Mineral Mix, S10026	10	0	10	0	10	0	10	0	10	0	10	0
DiCalcium Phosphate	13	0	13	0	13	0	13	0	13	0	13	0
Calcium Carbonate	5.5	0	5.5	0	5.5	0	5.5	0	5.5	0	5.5	0
Potassium Citrate, 1 H2O	16.5	0	16.5	0	16.5	0	16.5	0	16.5	0	16.5	0
Vitamin Mix, V10001	10	40	10	40	10	40	10	40	10	40	10	40
Choline Bitartrate	2	0	2	0	2	0	2	0	2	0	2	0
FD&C Yellow Dye #5	0	0	0	0	0	0	0	0	0	0	0	0
FD&C Red Dye #40	0	0	0	0	0	0	0	0	0	0	0	0
FD&C Blue Dye #1	0.0125	0	0.05	0	0.075	0	0.125	0	0.175	0	0.225	0
**Total**	**1064.6**	**4057**	**970.79**	**4057**	**923.83**	**4057**	**876.37**	**4057**	**782.83**	**4057**	**736.18**	**4057**
